# Design of Long-Life Wireless Near-Field Hydrogen Gas Sensor

**DOI:** 10.3390/s24041332

**Published:** 2024-02-19

**Authors:** Xintao Deng, Jinwei Sun, Fuyuan Yang, Minggao Ouyang

**Affiliations:** School of Vehicle and Mobility, State Key Laboratory of Automotive Safety and Energy, Tsinghua University, Beijing 100084, China; sjw@tsinghua.edu.cn (J.S.); fyyang@tsinghua.edu.cn (F.Y.); ouymg@tsinghua.edu.cn (M.O.)

**Keywords:** hydrogen detection, hydrogen safety, wireless sensor

## Abstract

A compact wireless near-field hydrogen gas sensor is proposed, which detects leaking hydrogen near its source to achieve fast responses and high reliability. A semiconductor-type sensing element is implemented in the sensor, which can provide a significant response in 100 ms when stimulated by pure hydrogen. The overall response time is shortened by orders of magnitude compared to conventional sensors according to simulation results, which will be within 200 ms, compared with over 25 s for spatial concentration sensors under the worst conditions. Over 1 year maintenance intervals are enabled by wireless design based on the Bluetooth low energy protocol. The average energy consumption during a single alarm process is 153 μJ/s. The whole sensor is integrated on a 20 × 26 mm circuit board for compact use.

## 1. Introduction

With the exploration of new energy, hydrogen has shown the potential to become the best energy carrier in the future low-carbon society. Hydrogen-related industries, including hydrogen production, the chemical industry, metallurgy, transportation, and energy storage are expanding and gradually moving towards large-scale applications.

However, hydrogen is a flammable and explosive gas with high energy density and a low explosion limit. Moreover, hydrogen gas molecules are so small that they can escape from tiny cracks and even penetrate materials. Therefore, the production, storage, transportation, and utilization system of hydrogen faces great safety challenges. In order to promote hydrogen applications and infrastructures, many demonstration projects have been carried out around the world, which results in the hydrogen-related facilities moving from being under centralized control and standardized operation to decentralized situations where the working conditions are unpredictable, and strict operational specifications are difficult to guarantee. As a result, safety issues and accidents follow. In most safety issues with different severity, hydrogen leakage acts as a critical link in the mechanism of failure. According to the H2Tools Database [1], in 220 reported hydrogen-related issues, 83 of them were related to hydrogen leakage, accounting for 37.73%. In fact, in other issues, hydrogen leakage is also very likely to be the cause, a potential consequence, or one of the links. Therefore, the hydrogen safety risk related to hydrogen leakage is worth paying extra attention to.

Efforts have been made in finding more sensitive, more stable, and faster hydrogen-sensitive materials and fabricating corresponding devices [2]. Currently, the most mature solutions include catalytic, electrochemical, semiconductor, and capacitive type sensing elements. These have been used on commercially available hydrogen sensors in a variety of scenarios. Optical and acoustic type elements are in the lab stage.

The sensing element alone is not enough for practical applications. It requires peripheral circuits, including power supply, signal processing, and signal transmission, and an appropriate system layout and installation to perform hydrogen detection. At present, the detection of hydrogen leakage in industrial and transportation scenarios takes place by installing sensors at the top of the space where the hydrogen system is located (referred to below as a spatial diffusion sensor). It is expected that leaking hydrogen will diffuse and accumulate around the sensor under a concentration gradient and buoyancy to trigger the alarm. However, this detection method may experience a very long delay, or even have no response to leakage that is disturbed by air flow, specific jet direction, and space shape, not only because the intrinsic delay of the sensor itself [3], but also because hydrogen may accumulate somewhere where it is undetectable, posing potential ignition or explosion risks [4,5]. In these situations, it’s critical to reduce the delay in hydrogen leak detection to provide enough time for emergency measures such as ventilation, system shut-down, or maintenance. This can be mitigated to some extent by installing distributed sensors throughout a space to form a sensor network. Wired sensors are not appropriate for network application, so previous research mainly focused on wireless sensors that implemented catalytic [6,7], semiconductor [8], and acoustic [9] type sensing elements. They used batteries as a power supply and transmitted data through RF or Zigbee and were still expected to be used as spatial diffusion sensors with a large size to carry batteries. There were also passive sensors that used radio frequency identification (RFID) technology to transmit data [10,11]. The RFID antenna was partially formed using a gas-sensitive material. When making contact with the target gas, the resonant frequency will change. But sensing distance has long been a problem of such sensors, which is normally ranges from centimeters to several meters. Optical sensors can also be used as distributed sensors, but are difficult to use as wireless sensors [12,13,14]. For fast hydrogen detection in outdoor facilities or the narrow cabins of hydrogen fuel cell vehicles, the aforementioned sensors still need improvements for actual implementation considering the limited space where they are installed and the cost of maintenance.

This paper proposes a long-life wireless near-field detection system aimed at allowing leaking hydrogen to contact the hydrogen sensor immediately to save on the time that passes while the hydrogen flows, diffuses, and accumulates. This is achieved by designing compact sensors mounted on pipe joints, as statistics showed that leakages in hydrogen systems occur primarily at threaded pipe fittings, including bite-type fittings, taper thread fittings, etc. [15,16]. The term ‘near-field’ means that the detection takes place at the location closes to the leakage. Wireless data transmitting and low-power consumption are achieved using Bluetooth Low Energy (BLE), which enables the sensors to be installed and distributed with lower maintenance and installation costs.

## 2. Near-Field Sensor Design

The near-field sensor was designed to be mounted on pipe joints while having a compact size. The housing can fit the nuts and be installed or removed without disconnecting the joint. After being installed, a confined chamber is created inside the sensor housing, allowing hydrogen released due to joint failure to enter it. So even tiny leakages, which often act as the pre-steps of more serious failures, will build up a highly concentrated hydrogen atmosphere that can be detected by the sensing element. In addition, the structure can also prevent interfering gas and dust in the environment from disturbing the sensing process.

For compact and wireless applications, it is important to pay attention to the power consumption of the sensing element and its peripheral circuit while maintaining satisfactory sensitivity and response times. Catalytic-type sensing elements broke the rule of low power, due to the need for continuous heating. Electrochemical-type elements were also excluded due to their slow response. Fiber-optic elements were not suitable for the scenarios being discussed due to the need for a physical connection of optical fibers and the high requirements for the light source and the receiver. Acoustic-type elements can be a low-priority candidate, for they require a complex and highly accurate peripheral circuit to excite the piezoelectric substrate and receive waves [17]. In the end, we decided that the preferred solution was a SMOX-type element based on the MEMS process, because it had all the features we needed, including a fast response, high sensitivity, low power consumption, and compact size. A commercially available sensing element (chip package, TGS8100 from Figaro) was used as the core element for detecting hydrogen. It was based on an N-type metal oxide semiconductor and was manufactured using the micro-electro-mechanical system (MEMS) process, which ensures its small size, low power consumption, fast response, and low detection limit [2,18].

**Figure 1:**
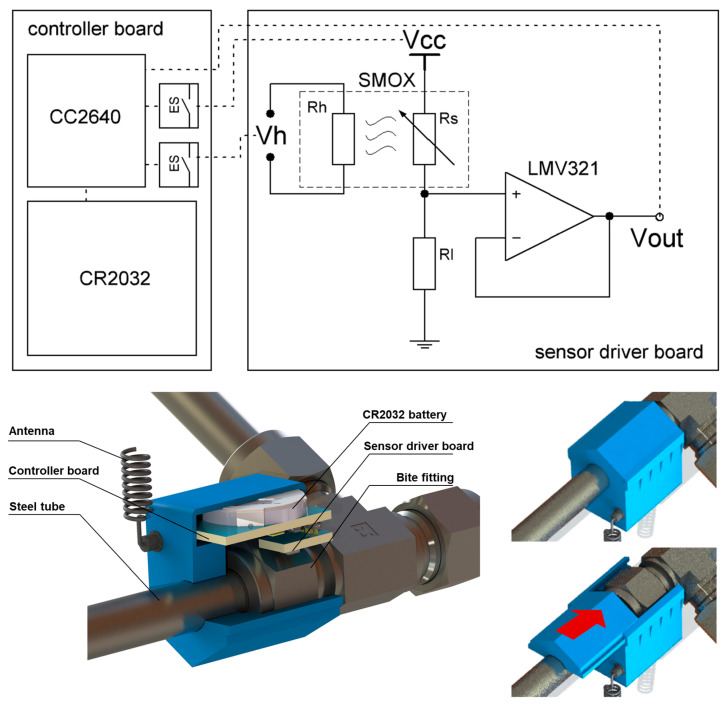
Electronic schematic and mechanical structure of the near-field sensor.

A peripheral circuit board was placed in the designed sensor housing with a CR2032 battery chamber. Then, the board was fixed and sealed with insulating glue to avoid possible sparks caused by short circuits and to protect the electronic components. The detection site of the sensing element was exposed to enable it to contact the gas inside the housing. A sectional view of the sensor is shown in Figure 1.

Wireless feature implementation was achieved based on the Bluetooth Low Energy (BLE) 5.0 protocol. An ultra-low power wireless MCU (CC2640 from Texas Instruments, Dallas, TX, USA) was used as the controller, signal processor, and data transmitter of the near-field sensor. The SMOX sensing element was controlled by the CC2640 using an electronic switch (ES). The output of SMOX was first fed into a voltage follower (LMV321 from Texas Instruments) and then collected by the CC2640.

## 3. Results

### 3.1. CFD Case Study

**Figure 2:**
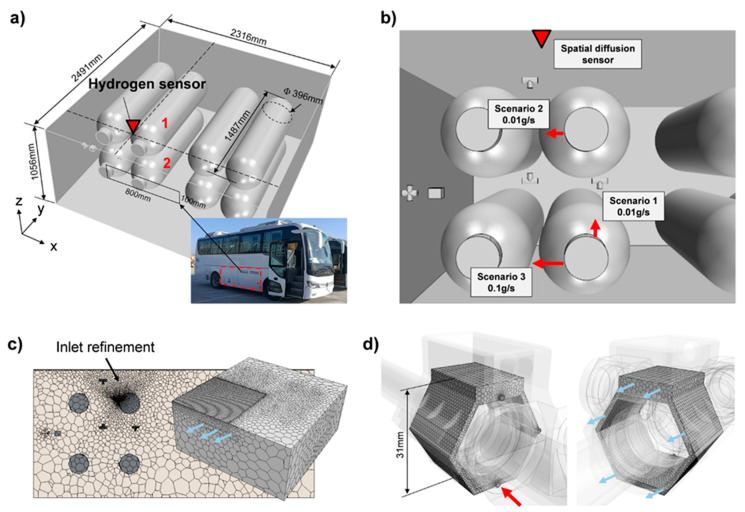
(**a**) Enclosure geometry; (**b**) scenarios schematic; (**c**) numerical grid for hydrogen tank cabin; (**d**) numerical grid for near-field sensor. Red arrows denote mass flow inlets, blue arrows denote pressure outlets.

To illustrate the advantages of near-field detection in reducing response times, we used the computational fluid dynamics (CFD) method to simulate and compare near-field detection with spatial diffusion detection devices in actual scenarios. The geometry used in the simulation was the hydrogen tank compartment of a hydrogen fuel cell bus, which is located at the bottom of the bus as shown in Figure 2a. We assumed that the leakage took place at the joints of the cylinder valves, forming a hydrogen jet flow. A spatial diffusion sensor was installed above the cylinder tanks and near the ceiling. The geometric specification was shown in Figure 2a. For near-field sensor, we built a model according to its housing as a CFD calculated region as shown in Figure 2d.

We selected three scenarios to assess the time taken from the start of hydrogen release to the occurrence of 1%vol hydrogen around the sensors. As shown in Figure 2b and Table 1, the leak points were chosen to be on the supply port of tank 2, the refill port of tank 1, and the refill port of tank 2, with a 0.01 g/s, 0.01 g/s, and 0.1 g/s mass flow rate, respectively. All leakages were considered to be related to pipe joint failures, and as such, they could be monitored by either the spatial diffusion sensor or the near-field sensors installed on the pipe joints. The inlet boundaries were modelled with a diameter of 1 mm and set as mass flow inlets. An opening of 800 × 100 mm was set as a 0 Pa pressure outlet on the cabin wall. The backside of a double ferrule joint was set as an outlet according to [16]. The positions of the leak points and the spatial diffusion sensor are shown in Table 1.

**Figure 3:**
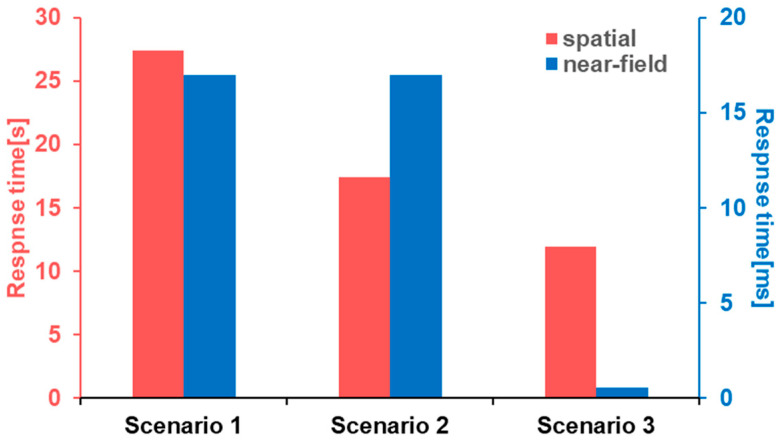
Response times of the spatial and near-field sensors in three scenarios.

**Figure 4:**
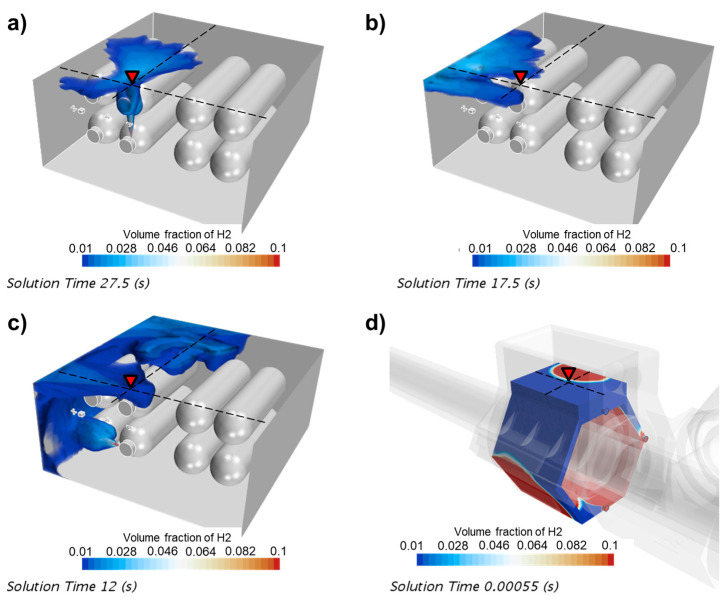
Cloud plot of leaking hydrogen in (**a**) scenario 1, (**b**) scenario 2, (**c**) scenario 3 and (**d**) the enclosure of the near-field sensor. The color bands are clipped to 1~10%vol hydrogen for better illustration.

A CFD model was calculated using the k-ε turbulence model. We used 100,000 mesh grids for the tank cabin model and 11,000 for the near-field sensor model. The response times and cloud plots were extracted for each scenario. Figure 3 shows that the near-field sensors will make contact with hydrogen within several milliseconds in all scenarios. This was expected, as the volume for leaking gas was extremely small. Compared to the near-field sensor, it took more than 10 s for the spatial sensor to be triggered. In scenario 1, the flow rate around the sensor was relatively low because the leak point was far away. Also, the upward jet flow was blocked by another tank, so the expansion of the hydrogen cloud at the top was slowed down. In scenario 2, although the sensor was close to the leak point, the jet flow direction was pointed in a horizontal direction, which caused the leaking hydrogen to move leftward while floating up, creating a hollow region around the sensor where convection and diffusion was weak, as shown in Figure 4b. This effect further slowed down the increase in the hydrogen concentration at the detection point. The mass flow rate was higher in scenario 3, but with a horizontal jet and other tanks blocking its way up, the response time was over 10 s and even more hydrogen was released compared to the previous scenes.

### 3.2. Near-Field Sensor Tests

**Figure 5:**
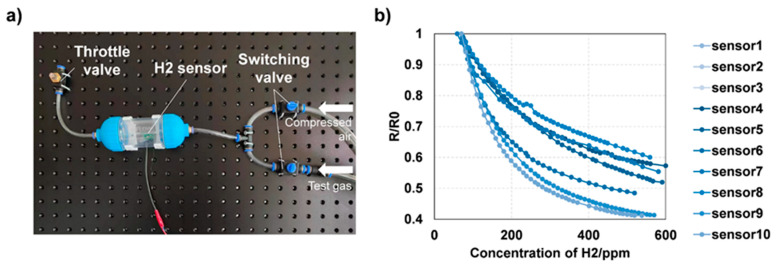
(**a**) Sensor test stand; (**b**) steady-state test results.

Steady-state response tests were carried out on ten near-field sensors at room temperature and ambient pressure using the test stand shown in Figure 5a. A premixed hydrogen–nitrogen test gas was supplied by Air Liquide^®^ (Paris, France). It should be pointed out that nitrogen mixing will not affect the response process of the sensor, because the process is dominated by a reductive gas like hydrogen, even under a low concentration. The response-concentration curve in Figure 5b shows that the sensor can produce a significant response at 0~500 ppm of hydrogen. When the concentration exceeds 200~300 ppm, the sensitivity of the sensor begins to decrease and becomes saturated at 500 ppm.

For an N-type semiconducting metal oxide (SMOX) material, such as the SnO_2_ and ZnO used in our work, the adsorption of oxidizing gases such as oxygen will establish an electron depletion region at the material’s surface, which decreases the overall carrier concentration causing the material’s conductivity to decrease. Conversely, if reducing gases such as hydrogen are adsorbed, the carrier concentration will increase causing the conductivity to increase [19]. The sensing process generally needs heating to accelerate the surface reaction. The SMOX particles manufactured using the MEMS process are very tiny, so they are highly sensitive and require low heating power, but also have low saturation concentrations. The responses varied between the sensors, although the parameters and environmental conditions were the same. This may have been caused by the lack of consistency in manufacturing, such as differences in SMOX particle size, surface morphology and total layer thickness. Since our sensors were meant to signal hydrogen leakage rather than measure the hydrogen concentration, our design only provided “0–1” responses instead of analog results.

**Figure 6:**
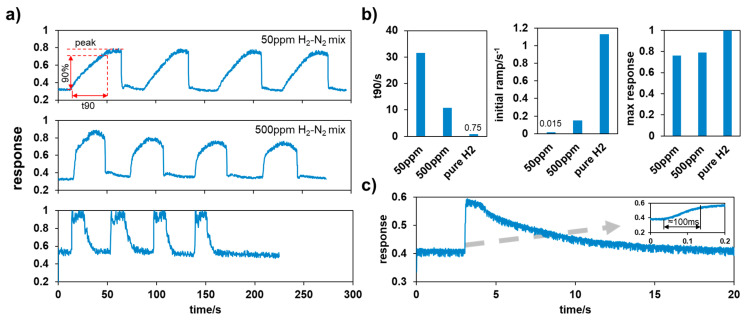
(**a**) Dynamic test results; (**b**) response characteristics of different test gases; (**c**) sensor response to a single triggering by pure hydrogen.

Dynamic tests were also carried out using the test stand, which involved multiple cycles of test gas and compressed air being released into the sensor chamber in turns. The upstream pressure was 0.5 bar, and the flow rate was 800 L/min. According to the dynamic test results shown in Figure 6a, the responses to different hydrogen concentrations had different characteristics, including curve shapes, ramp rates, and maximum values. Figure 6b summaries these factors, showing that the initial ramp rate and maximum response increased, while the time it took for the signal output to reach 90% of its peak (t90) declined with increasing concentrations. This was mainly because of different adsorption rates under different ambient concentrations. Considering the unsatisfying t90, the initial ramp rate should be used as the alarm trigger instead of the absolute value of the response. As shown in Figure 6c, the initial ramp of the sensor stimulated by pure hydrogen was dramatic and fast enough to meet our detection requirements.

**Figure 7:**
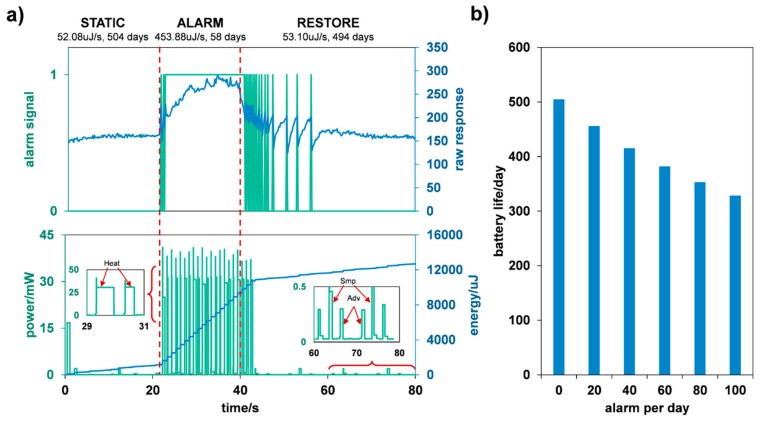
(**a**) Signal output and energy consumption during a triggering process of the wireless sensor. The green line in the upper figure stands for the 0-1 alarm signal which was generated according to the sensing element output by CC2640, the blue lines stands for the original response signal of sensing element. The detailed power peak is shown in subplots, where heat stands for heating, smp stands for ADC sampling, adv stands for advertisement; (**b**) estimated battery life with different numbers of alarms per day. The duration of each alarm was estimated to be 1 min.

**Figure 8:**
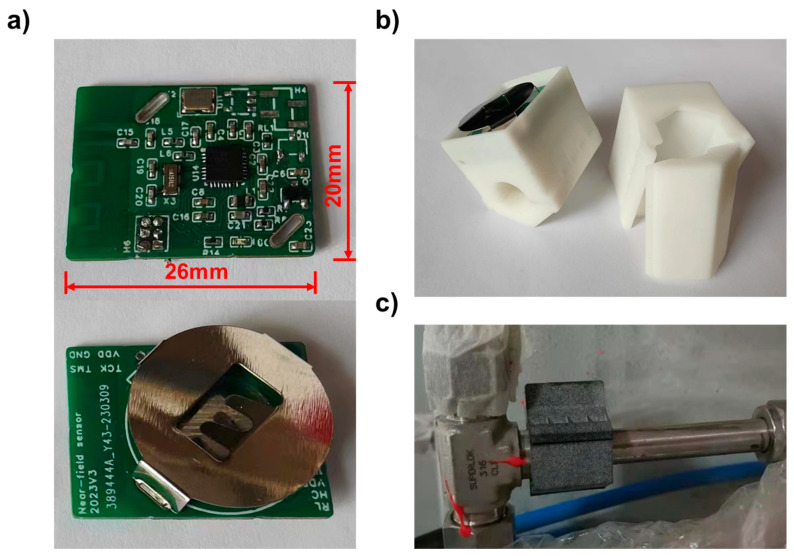
(**a**) Wireless MCU unit; (**b**) near-field sensor with 3d printed housing; (**c**) installation of the sensor.

After assembling the whole sensor as in Figure 1, we implemented a low power control strategy. Alarm signals and other information such as estimated battery life were transmitted using non-connectable advertisement by the CC2640 every few seconds. The heating was turned off at the static state (no gas leak detected) to remain in low-power mode. Once triggered by hydrogen, the sensor will enter an alarm state with pulsed heating and more frequent advertisement. After the leakage, the heating continued to help the sensor restore to a static state. The whole triggering process with a low power control strategy is shown in Figure 7a. Around 53 μJ/s of energy consumption was achieved under the static state, which resulted in a lifetime of over 500 days with one 210 mAh CR2032 battery. In the alarm state, the energy consumption was about nine times higher than in the static state, and during a single alarm process, as shown in Figure 7a, the average energy consumption was 153 μJ/s. The estimated lifetime is shown in Figure 7b with different numbers of alarms per day. An assembled wireless near-field sensor was shown in Figure 8. The circuit board was designed in the size of 20 × 26 mm, so that the sensor can be installed on 3/8” bite-type pipe fittings as in Figure 8c.

## 4. Conclusions

In this paper, a compact wireless near-field hydrogen sensor was designed, which is aimed at detecting pipe joint leakages. CFD results showed that the time taken for a near-field sensor to react to hydrogen leakage was shortened by orders of magnitude in all three scenarios, reducing the risks brought by the accumulation of flammable mixtures. The wireless feature was implemented using the BLE 5.0 protocol and achieved a battery lifetime of over 1 year with one CR3032.

The next step is to assess the safety of the implementation of the near-field sensor, since it contains a battery and wireless transmission elements. We are optimistic about achieving intrinsic explosion-proof safety based on the low power characteristics of the near-field sensor. In actual applications, near-field sensors may not be necessary for all joints in a system. We can give full play to the advantages of the wide coverage of a spatial concentration sensor, letting it monitor potential leakage locations with a large hydrogen mass flow, appropriate jet flow direction, and fewer obstacles in the diffusion path, while using near-field sensors in locations that are difficult to be covered. In this way, the safety of the system will be guaranteed, and costs can be optimized. The design of a hybrid detection system deserves further study.

## Figures and Tables

**Table 1:** Coordinates of the leak points and the sensor.

Subject	Coordinate	Jet Direction	Mass Flow Rate
Scenario 1	(835, 500, 353)	z+	0.01 g/s
Scenario 2	(706, 490, 778)	x−	0.01 g/s
Scenario 3	(706, 490, 278)	x−	0.1 g/s
Spatial diffusion sensor	(694, 300, 1023)	/	/

## Data Availability

The data presented in this study are available on request from the corresponding author. The data are not publicly available due to restriction of research institute.
